# Daylight Sonography: Clinical Relevance of Color-Tinted Ultrasound Imaging

**DOI:** 10.3390/life15111672

**Published:** 2025-10-27

**Authors:** Christoph F. Dietrich, Matthias Wüstner, Christian Jenssen, Daniel Merkel, Jörg S. Bleck

**Affiliations:** 1 University Hospital Frankfurt, Johann-Wolfgang-Goethe University Frankfurt, D 60590 Frankfurt/M., Germany; 2 Central Interdisciplinary Sonography, Krankenhaus der Barmherzigen Brüder Trier, D 54942 Trier, Germany; m.wuestner@mail.de; 3 Brandenburg Institute for Clinical Ultrasound (BICUS) at Brandenburg Medical University, D 16816 Neuruppin, Germany; 4 Department for Internal Medicine, Krankenhaus Märkisch Oderland, D 15344 Strausberg, Germany; c.jenssen@khmol.de; 5 Department for Internal Medicine, Caritas-Klinik Dominikus, D 13467 Berlin, Germany; daniel.merkel@mhb-fontane.de; 6 Ultrasound-Division, INI—International Neuroscience Institute Hannover, D 30625 Hannover, Germany; joergsbleck@gmail.com

**Keywords:** ultrasound, sonography, photopic vision, color scales, daylight conditions

## Abstract

Daylight sonography refers to the technique of using color-tinted, brightness and contrast-controlled B-mode ultrasound images optimized for well-lit environments. Originally introduced as Photopic (“Tageslichtsonographie”, Daylight sonography) by Siemens, this approach addresses the limitations of grayscale imaging under ambient light conditions. With the growing emphasis on point-of-care ultrasound (POCUS) and mobile diagnostics, daylight-compatible imaging modes such as Photopic Mode have become increasingly relevant since they are performed under daylight conditions. This paper explores the technical background, visual physiology, clinical applications, implementation challenges, and future perspectives of daylight sonography, advocating for broader use and standardization. Standardization, further clinical validation, and integration with emerging technologies such as artificial intelligence (AI) are essential to fully realize the potential of daylight sonography in routine practice.

## 1. Introduction

Over the past five decades, grayscale B-mode imaging has become the standard in diagnostic ultrasound. However, conventional grayscale displays are optimized for scotopic (dim-light) vision, typically requiring darkened rooms for optimal contrast perception. This poses challenges in modern clinical environments where point-of-care decisions, mobile imaging, and rapid workflows are necessary. Daylight sonography using brightness-coded color-tinted images has emerged as a solution to enable ultrasound imaging under photopic (daylight) conditions [1,2,3,4,5].

The idea is rooted in visual physiology: under bright lighting, cone cells in the retina, responsible for color vision and high-resolution perception, dominate visual processing. Therefore, converting gray values into color gradients enhances the visibility of anatomical structures without changing the underlying diagnostic data.

Not only light intensity but also the spectral quality of ambient light influences perception. For example, cool (blue-enriched) lighting enhances alertness and favors the perception of blue and green hues, while warm lighting (yellow/red spectrum) reduces visual acuity for these tones.

Visual differentiation of tissue in low-contrast organs has been a problem since the early days of ultrasound imaging and improved spatial (geometric) and contrast resolution has been a key goal of ultrasound technology.

### Aim

This paper aims to explore and establish the clinical relevance of daylight sonography, a technique that utilizes color-tinted, brightness and contrast-coded ultrasound imaging optimized for photopic (daylight) conditions.

Specifically, this paper seeks to

- Explain the visual physiological basis for adapting ultrasound imaging to photopic vision, highlighting the limitations of conventional grayscale B-mode in well-lit environments.
- Review the historical and technical evolution of color-coded ultrasound imaging, with an emphasis on Look-Up Table (LUT) development and photopic display algorithms.
- Assess the clinical applications of photopic ultrasound across emergency, interventional, outpatient, and mobile settings—where ambient light conditions challenge traditional imaging workflows.
- Identify common artifacts and optimization strategies related to photopic imaging, including adaptive color scaling and artifact suppression.
- Present user experience data from a pilot study comparing preferences between grayscale and photopic modalities.
- Propose integration with artificial intelligence (AI) to support real-time optimization, standardization, and interpretation of color-coded images.
- Discuss educational, ergonomic, and practical implications, including training needs and potential improvements in visual fatigue and workflow efficiency.
- Outline future directions for research, standardization, and clinical validation of daylight-compatible ultrasound imaging.

## 2. Visual Physiology and the Need for Colorization

The human retina comprises two types of photoreceptors: rods and cones. Rods function under low-light (scotopic) conditions and are sensitive to grayscale contrast but offer low resolution. Cones, by contrast, operate in bright-light (photopic) conditions and enable color discrimination and high spatial acuity. At the center of the retina, the fovea centralis has the highest density of cones (over 100,000 per mm^2^), making it ideal for high-detail color perception; examples include daily applications [6,7,8,9,10,11,12,13,14,15,16,17].

Traditional B-mode ultrasound converts echo intensities into gray levels. Under daylight conditions, the eye’s ability to differentiate these shades of gray diminishes. By transforming grayscale values into carefully calibrated color gradients, daylight sonography enhances image interpretation in bright clinical settings. In the color range, up to 16,777,216 colors can be created by varying the red, green and blue channels with 256 levels (8 bits). The eye can distinguish over 10,000 shades of color but only 15–60 grayscale levels under dim light [18]. This stark difference justifies the shift to color-coded imaging in photopic environments [6,7,8,9,10].

An additional consideration is the difference between static images and moving ultrasound sequences. It is well established that moving images enhance the perception of subtle gray-level differences due to temporal integration in visual processing. However, under bright ambient light, grayscale differentiation is still markedly limited. Photopic imaging improves visibility in both static images and moving sequences by expanding contrast perception and enhancing 3D impression. This effect is particularly relevant in daylight conditions, where color-tinted images allow reliable recognition of lesions and anatomical structures that may otherwise be overlooked on conventional grayscale displays.

## 3. Technical Foundations and Evolution

The earliest effort to colorize ultrasound images dates to the 1960s and 1970s, using photographic filters and early digital methods [19,20]. These attempts often relied on discrete, limited-color palettes that introduced interpretation artifacts. For example, using eight to sixteen arbitrary color tones often led to poor correlation between echogenicity and perceived structure [21,22].

Significant improvements came with continuous color scales such as the annealing scale by Milan and Taylor [23] and the gradient model by Chan and Pizer [24], which allowed real-time dynamic color mapping of echogenicity differences. Modern ultrasound platforms implement these techniques using real-time digital image processing and adaptive Look-Up Tables (LUTs), which transform grayscale histograms into perceptually optimized color displays with static functionality *(B-color mode)* [25], while “photopic” mode was based on dynamic histograms.

B-color and photopic modes are distinct technologies. B-color applies fixed color conversions, often causing artifacts like brightness imbalance or color saturation errors. Photopic imaging, however, uses histogram analysis, adaptive algorithms, and a limited range of hues (e.g., sepia, green, blue) to enhance contrast while minimizing artifacts [1,26,27,28].

After the introduction of color photography in everyday life in 1967, Adams and Jeffe photographed ultrasound images from a monitor through superimposable filters of different colors to create a color impression. The process was time-consuming due to the limited number of colors, the color filters used or their superimposition and the entire procedure [19]. A three-color coding scheme was used to match the red/green/blue color channels [20]. This enabled elegant and simple coloring in LUTs.

**Figure 1:**
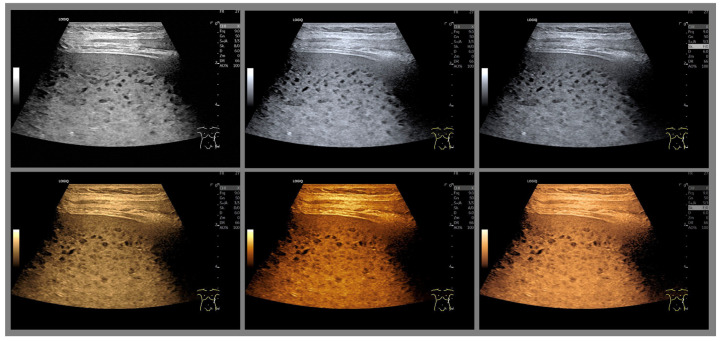
Enlarged spleen with multifocal small hypoechoic parenchymal nodules in an 87-year-old female patient with malignant lymphoma, imaged with 3 different gray map variants and 3 different yellow/brown tones on a GE E10 ultrasound machine (GE HealthCare Technologies, CA, USA).

**Figure 2:**
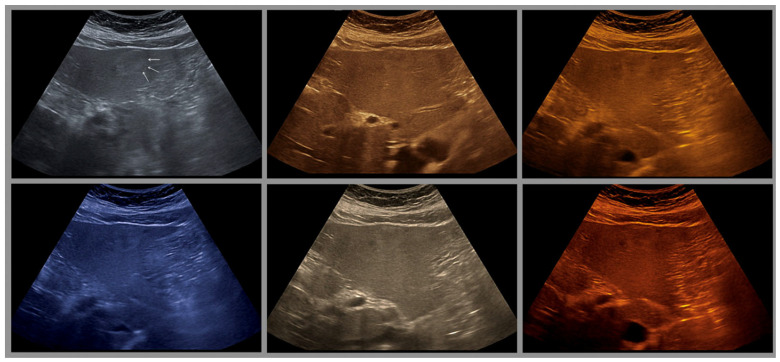
Examples of an isoechoic focal liver lesion (arrows) in a 43-year-old female patient, imaged with 6 different color variants on a Fujifilm Arietta 850 (Fujifilm Corporation, Tokyo, Japan). The differences in the sectional image are the result of slightly different transducer positions and different breathing positions of the patient.

Several years later, in 1976, Baum used twelve different color scales for coloring [29]. Since that time (1975), multicolored color scales of 8–16 color levels have been used to arbitrarily assign colors to echo signals. The introduction of the two-color glow color scale in the red-brown range by Borcke in 1983 led to artifact-free image enhancement for the first time through a continuous color gradient. This can probably be considered the basis for the yellow-brown coloration that is very popular in most ultrasound devices today [30]. Currently available tint-colored images are shown in (Figure 1 and Figure 2).

In radiology at this time, black and white findings of scintigraphy with radioisotopes were photographed and displayed through colored filters [31,32,33,34]

The spectra-color method [35,36,37,38,39] allowed different frequency ranges of an ultrasound image to be displayed in different colors; the working group around Yokoi and Ito used this method in the so-called C-mode and ultrasound tomograms [40,41,42,43,44,45,46,47,48]. In these studies, the authors predominantly reported good results for improved structural recognition of tumors. However, there were also obvious disadvantages, as the arbitrary color assignment had to be learned first, in contrast to the intuitively understandable brightness-coded analyses of black-and-white or gray-scale images. For example, green or reddish tones are assigned to lighter areas and bluish tones to darker color areas, which is not immediately obvious. The differences in echogenicity that are easily recognizable and quantifiable in the gray image can sometimes only be perceived to a limited extent due to differences in color. The large color difference registered by the eye is in discrepancy to the originally small echogenicity difference. The sharp separation of color levels can also result in color artifacts caused by two directly adjacent dots with different color assignments.

The limited number of colors used (eight to 16) also reduces the image information, as the eye can perceive at least 30 different gray levels in the moving image [22]. Nevertheless, the cited published literature reported improved resolution and improved recognizability of small differences in echogenicity [49].

A significant improvement was achieved in the following years after 1976 by Chan and Pizer. Instead of discrete color spectra, a continuous color spectrum was used. The most widely used color scale was the annealing color scale introduced by Milan and Taylor in 1975 [23]. Continuous echogenicity gradients were translated into continuous color gradients from white-yellow to orange-red to brown-black [24].

Continuous color coding was subsequently established, although sometimes with different color compositions and scales [25,30,50,51,52]. By continuously assigning echogenicity differences to colors, the full information content of the sonogram is retained and echogenicity-related artifacts as well as the mental conversion from gray values to colors can be avoided [22,50]. This form of dynamic color coding can also be used in ultrasound devices in real time. However, the image and color result is not predictable [22], so that an image colored with the glow color scale, for example, only contains color valences of the red-green range, but the rest of the color scale remains unused. The quality of the sonogram is therefore generally poorer and this function is therefore rarely or never used.

### Scotopic Vision and Shades of Gray

**Figure 3:**
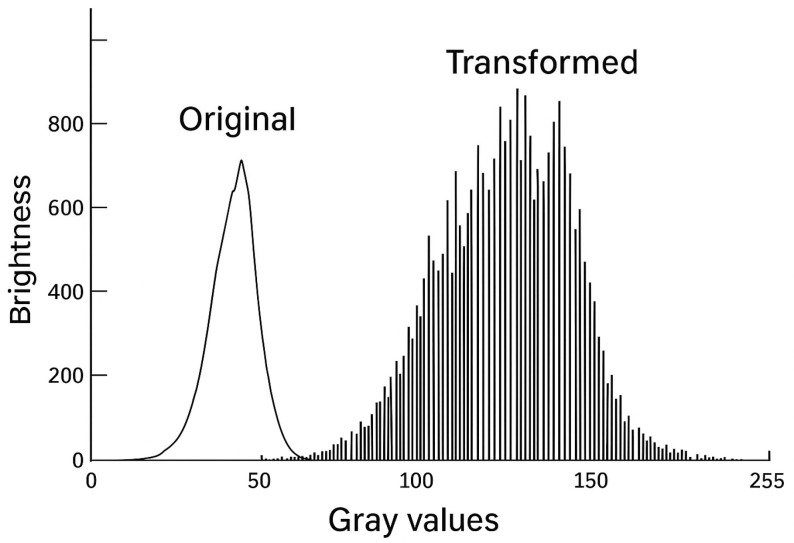
Histogram of an ultrasound image with gray values. The histogram of an ultrasound image is effectively its fingerprint, showing the frequency of gray values in each image. The average brightness is at the lower end of the available gray scale. The contrast range is low, as the eye is not very efficient in twilight vision (**left part**). The available gray scale (from 75 dB) is not used to its full extent. Two-stage solution with photopic ultrasound imaging (**right part**). First, the gray scale of the original image histogram is adaptively converted to the optimal visual range for daylight vision (right component). Each gray value is converted individually in order to achieve a more uniform distribution of gray values. The contrast range increases significantly with improved spatial resolution and improved depth perception.

**Figure 4:**
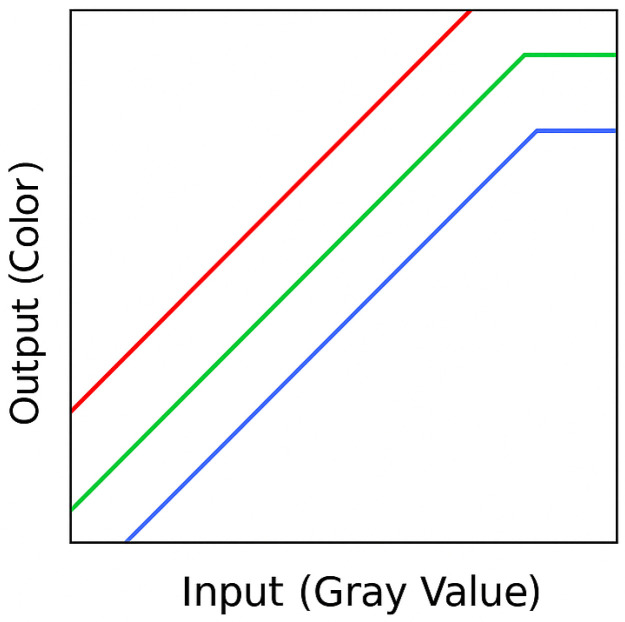
Red, green, blue (RGB) conversion for each gray value. This means that each gray value is displayed in a defined manner as a uniform (monochromatic) color tone.

In scotopic vision, i.e., the adaptation of visual perception in dark rooms, only a small gray scale range of 15 to 60 levels can be distinguished [21,22,50] with higher sensitivity in a moving image compared to a static image and depending on background luminance, adaptation and alertness. The amplitude of gray-scale information in an image is at the lower end of the gray-scale curve, where the eye is least able to resolve details (Figure 3 and Figure 4).

Photopic imaging is optimized for bright ambient conditions. In scotopic settings, where rod photoreceptors dominate, grayscale images may still provide superior differentiation of low-contrast structures. Thus, the advantage of photopic imaging is context-dependent: highly beneficial in daylight, but not necessarily superior in darkened environments.

Recent work suggests that the just noticeable difference (JND values) of the human eye for gray values can be up to 1000 gray levels under optimal luminance conditions [53].

Nowadays, a high-resolution ultrasound image usually contains up to 256 gray levels in a digital image (eight bits for the corresponding color representation) and up to 512 gray levels in individual devices [54]. There is therefore a discrepancy between the range of information provided by the ultrasound device on the one hand and the limited possibilities for perception by the examiner on the other.

In addition to ambient light, factors such as monitor technology, screen size, and display resolution influence human visual perception and thus the interpretation of ultrasound images. Larger monitors with higher pixel density and modern display technologies (e.g., OLED, HDR-capable panels) can enhance color fidelity and contrast perception, while smaller or lower-quality displays may reduce the effectiveness of photopic imaging. Although these aspects were not the primary focus of our review, they represent important variables that should be considered when implementing daylight-compatible ultrasound systems in clinical practice. Standardization efforts, such as those proposed by Kovesi [55] or by the International Electrotechnical Commission (IEC; EN 62563-1; IEC EN 62563-2), may also help to harmonize display requirements across manufacturers and ensure reproducible visual performance.

## 4. Clinical Implementation and Significance

Daylight sonography since its early beginnings has gained traction in various clinical settings [20,21,22,23,24,25,29,30,35,37,38,40,42,44,46,47,49,50,51,52,56,57,58]:

- The use of Handheld Ultrasound Devices, e.g., in the emergency and intensive care setting. POCUS is increasingly performed in uncontrolled lighting environments. Photopic imaging allows reliable assessment without requiring dark rooms [59]. POCUS examinations typically focus on questions that can also be adequately clarified with ultrasound devices with only moderate image quality, e.g., handheld ultrasound devices. This is particularly the case when the findings to be assessed are represented by large impedance differences in the ultrasound image (detection of intracavitary fluid, hydronephrosis or filled urinary bladder). In those cases, the advantages of photopic imaging are obvious, whereas the discrimination of small differences in echogenicity and B-mode ultrasound quality is less important.
- Interventional Procedures: Enhanced tissue and needle visibility in color-tinted images improve safety and accuracy in ultrasound-guided interventions.
- Outpatient and ambulatory Care: Eliminating the need for darkened rooms enables better integration of ultrasound into routine patient care.
- Supervised ultrasound training: Hands-on training and live demonstrations require daylight conditions so that trainees can observe how the trainers’ right hand handles the probe and what his left hand does with the knobs and buttons of the US machine.
- Lecturing: Colorized ultrasound images do not need a dark background, nor does the room need to be as dark as possible, which helps prevent the audience from losing attention.

Ergonomically, daylight sonography reduces eye strain by matching image contrast to ambient light. It also aligns with sonopsychology principles, creating a more welcoming environment for patients and supporting effective communication during examinations [60,61].

The authors have applied photopic imaging in daily practice, particularly during point-of-care ultrasound, interventional procedures, and teaching sessions. Based on this experience, color-tinted modes facilitate workflow in bright environments, improve the visibility of interventional needles, and enhance the teaching atmosphere by allowing both examiner and learners to maintain visual contact without dimming the room.

## 5. Artifact Management and Optimization

Colorized ultrasound images, while beneficial, are susceptible to specific artifacts if improperly calibrated. Three common artifact types are

- Hole artifacts: Echopoor pseudolesions as a result from clustering dark gray levels.
- Echogenicity artifacts: Local glare as a result of over-enhanced brightness from grouped light gray levels.
- Veiling (obscuration or homogenization) artifacts. Mid-gray clustering that reduces depth perception.

Artifact suppression relies on adaptive LUTs that adjust contrast and brightness based on real-time histogram analysis. Color maps with minimal saturation and linear hue transitions (e.g., sepia or muted green) reduce visual noise and preserve diagnostic detail. The Siemens SONOLINE Elegra platform was among the first to integrate these functions. Its algorithm allowed clinicians to adjust image brightness (h) and contrast (k) in real time. It also permitted region-of-interest (ROI) analysis for localized photopic enhancement [1].

## 6. Visual Adaptation and Practical Implications

In clinical reality, examination environments may alternate between dimly lit and bright conditions, such as when moving from dedicated ultrasound rooms to wards, emergency units, or outpatient clinics. These fluctuations can impair visual performance, as switching between scotopic and photopic conditions requires a period of visual adaptation. Photopic ultrasound imaging offers a practical advantage in such settings, since it can be performed consistently under daylight conditions, thereby avoiding adaptation delays and maintaining diagnostic efficiency. Comparative data indicate that although full dark adaptation may take up to 45 min [10], the majority of visual recovery occurs within the first few minutes; nevertheless, even this short delay can be disruptive in time-sensitive workflows. By reducing the need for repeated adaptation, daylight sonography supports both examiner comfort and workflow continuity.

Photopic imaging eliminates this delay. Ultrasound examinations can proceed under consistent lighting, improving efficiency and reducing cognitive load. This is especially important in

- Mobile units where lighting control is limited.
- Bedside diagnostics during hospital rounds.
- Triage and field settings in disaster or military medicine.
- Human Factors and Examiner Preferences.

Experienced examiners who have long relied on grayscale imaging may initially find it challenging to adapt to color-tinted displays. To facilitate acceptance, gradual integration of photopic imaging during teaching sessions and hands-on training can be helpful. Customizable LUT profiles that reflect familiar brightness-to-echogenicity mappings may also support the transition. Our pilot study further indicates generational differences: while most senior examiners preferred grayscale, medical students and younger examiners showed a stronger preference for photopic modes. These findings suggest that tailored training and flexible display options can bridge the adaptation gap and improve overall acceptance.

**Figure 5:**
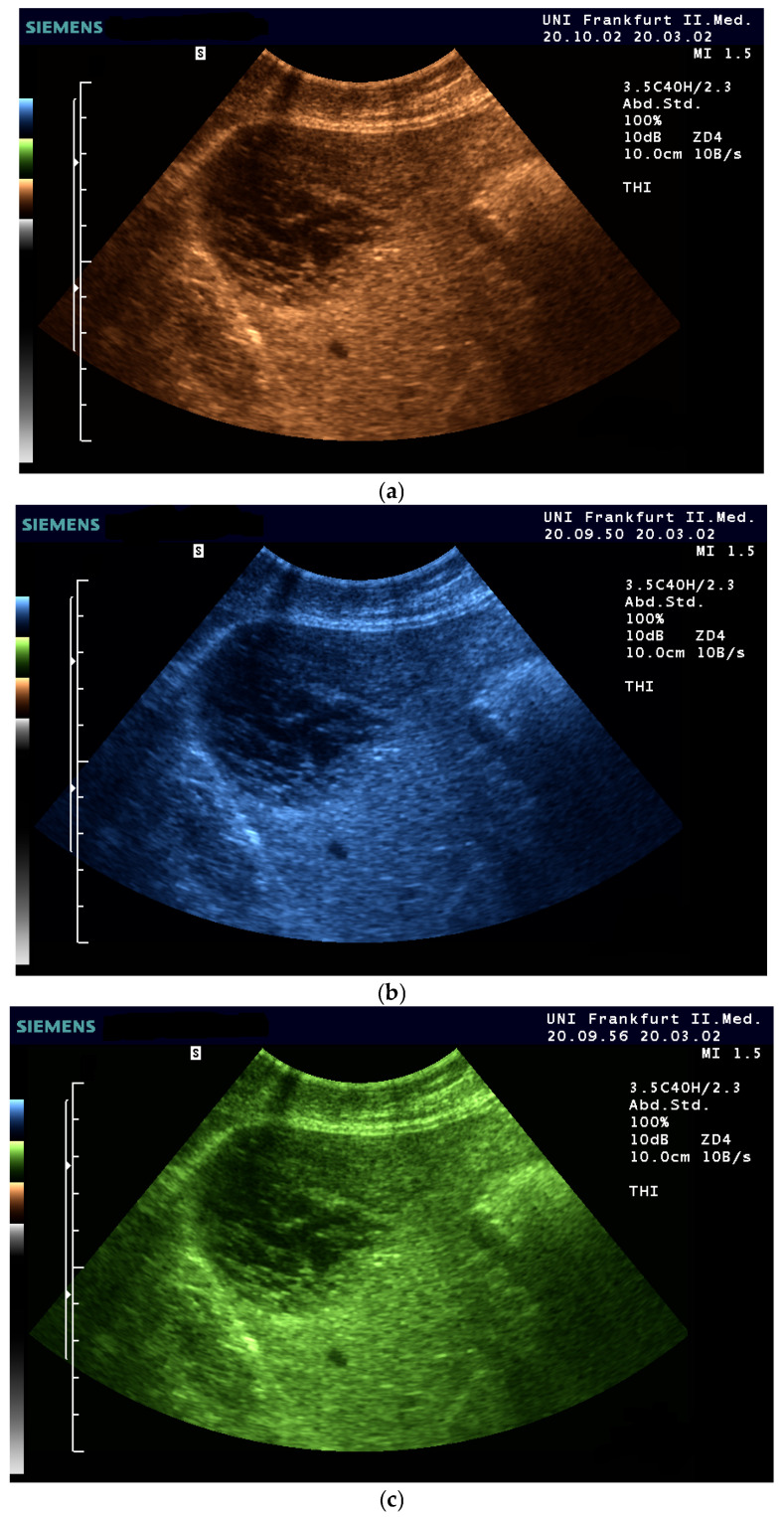

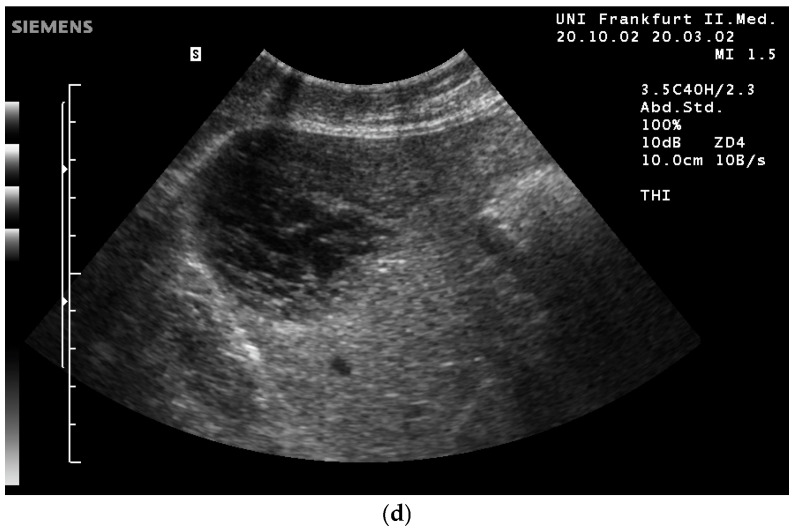
Examples of the comparison of colors using photopic in the mentioned study for experts and students. Artifact-free representation of a liver sarcoma at the same point in time using the four available photopic colors sepia (**a**), blue (**b**) and green (**c**) as well as gray (**d**). Saturation and intensity differ. Relevant factors influencing the photopic transformation of image information include image brightness, color type and color saturation. In order to realize a sonography in daylight, it is necessary to increase the image brightness and to increase the contrast as much as possible. All steps must be realized without artifacts.

**Figure 6:**
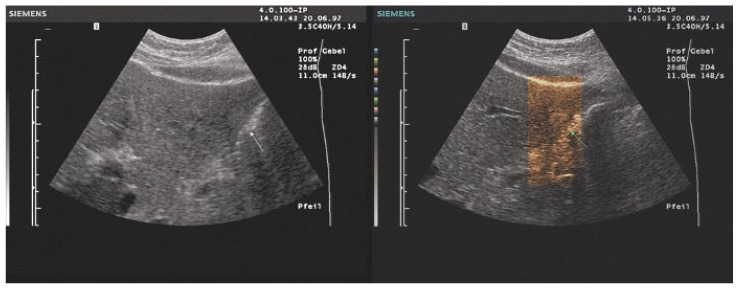
The visibility of a small liver hemangioma (arrow) is improved using a photopic display (Sepia, within the region of interest).

**Figure 7:**
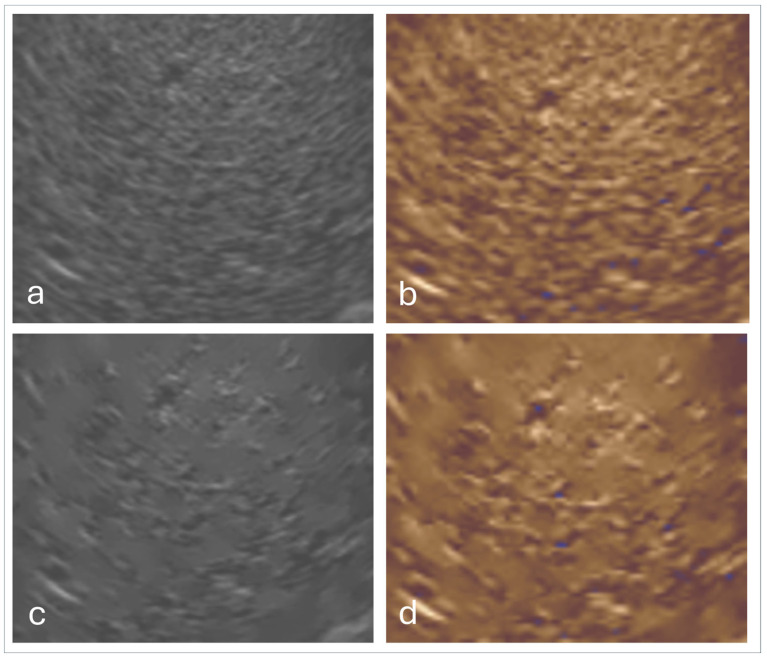
Sonographic liver texture in a patient with chronic hepatitis B visualized with standard greyscale imaging (**a**). Photopic imaging (Sepia) leads to significantly more vivid detail recognition (**b**). The remodeling signs of hepatitis, highlighted with a speckle algorithm (**c**), are also better visualized after Photopic (**d**).

A (not yet published) pilot study with 25 ultrasound experts and 25 medical students found generational differences in imaging preferences using true Photopic. Experienced examiners (83%) preferred grayscale, citing familiarity and diagnostic trust. However, most students (75%) favored color-tinted images, likely due to reduced bias and better visual engagement. Among color options, sepia was preferred by 67% of students and equally favored with blue by experienced examiners for better visualization (Figure 5, Figure 6 and Figure 7) [62].

### Is There an Ideal Color Map?

There is no convincing scientific data on which color map is preferable in general or for specific issues [58]. The study by Fischer et al. showed a slight advantage of a reddish-brown color scale over gray, blue, and green scales [63].

The various nuances of the yellow-brown color sepia are known from black and white photography. UV radiation and chemical processes based on sodium hydroxide and other factors can turn the black parts brownish and the white parts yellow to cream-colored. In modern photography with digital cameras, there are also special modes that achieve a sepia tone. This suggests that color perception varies individually and may require tailored settings for optimal use. Training and customizable LUT profiles may bridge the generational gap and improve acceptance [1].

Color perception is not only physiologically but also culturally influenced. For example, certain hues may be associated with positive or negative connotations depending on historical or regional context. These aspects should be taken into account when designing color-tinted ultrasound displays. Ideally, future ultrasound systems will allow users to freely customize Look-Up Tables (LUTs), enabling adaptation of color combinations to individual preference, cultural background, and specific diagnostic needs.

While broad categories such as red, green, or blue are often used to describe color schemes in ultrasound imaging, artistic and perceptual sciences highlight that each hue encompasses a wide spectrum of variations. For instance, ‘red’ can range from deep wine-red to light pastel shades, each eliciting different visual impressions. Such nuances are equally relevant in ultrasound imaging, where extreme saturation or high-contrast tones may exaggerate artifacts, whereas intermediate or muted variants (e.g., sepia, brownish-yellow, or desaturated blue-green) can enhance diagnostic clarity without overwhelming the observer. The use of continuous or intermediate color gradients in LUTs therefore represents a promising approach, bridging aesthetic principles from art and cinema with the practical requirements of clinical imaging.

Neurophysiological studies consistently show that exposure to blue light facilitates alertness and enhances performance on tasks requiring sustained attention [64,65,66,67,68,69]. Psychological studies also favor blue color scales, as blue is generally associated with trustworthiness, while data suggests that red evokes avoidance motivation and undermines intellectual performance in the Western World [70,71,72]. A different view is true for Asian countries compared to the Western World as shown in the discussion for elastographic imaging with respect to define stiffness by colors using red or blue (hard, soft) [73].

In contrast, high-saturation or arbitrarily colored LUTs (e.g., red hues) can lead to misinterpretation due to exaggerated color differences that do not correlate with real echogenicity differences. Red may introduce visual distortions, poor contrast mapping, and increased artifact risk, especially in detailed soft tissue imaging. The human eye has a relatively low sensitivity for red colors in bright conditions, whereas under photopic (daylight) conditions, the eye’s cone cells are most sensitive to green and blue wavelengths. Red light is less sharply perceived, especially in low-saturation forms, leading to poorer contrast discrimination. This means red-tinted areas may appear washed out or less detailed, particularly for subtle structures. In visual art and perception science, red is a ‘warm color’ that psychologically advances (i.e., appears closer). This can distort depth perception in ultrasound, making superficial structures seem artificially prominent and deeper ones harder to distinguish. Red tones are easily oversaturated, leading to artifacts such as glare, blooming, or artificial echogenicity spikes. High saturation in red may mask subtle gray-level differences, reducing diagnostic accuracy. In adaptive Look-Up Tables (LUTs), red does not transition as smoothly to other hues (compared to sepia or green), increasing the risk of color banding or abrupt brightness shifts.

Therefore, red is generally avoided in daylight sonography, where sepia, blue, and muted green provide more stable and diagnostically useful color gradients (Table 1).

## 7. Integration with Advanced Ultrasound Modalities

Photopic imaging complements advanced imaging methods like

- Tissue Harmonic Imaging (THI): Enhances resolution by reducing noise and improving border delineation [26,63,74,75].
- Contrast-Enhanced Ultrasound (CEUS): When combined with photopic imaging, CEUS benefits from improved brightness perception and reduced artifact interference.
- Photopic imaging may also hold promise for Doppler (including spectral FFT analysis) and elastography (e.g., 2D-SWE). By improving contrast perception under bright ambient light, photopic display modes could facilitate the detection of subtle vascular signals or enhance interpretation of elastographic stiffness maps. However, current implementations are limited, and systematic studies evaluating the diagnostic impact of photopic imaging in these modalities are lacking. Further research is therefore required to validate its clinical usefulness and to establish standardized settings.
- The combination with speckle filters available in all high-end devices, similar to the low-pass filters used by Bleck et al. (1994) for tissue visualization [5], also yields significantly improved visualization results (Figure 5, Figure 6 and Figure 7). In principle, applications with new visualized texture analyses, such as random field models, are also conceivable [76,77].

Photopic settings allow the use of lower transmit power (important in fetal and ocular ultrasound), reduced mechanical index (MI), and thermal index (TI), while maintaining contrast resolution and diagnostic clarity (Figure 7) [78].

## 8. Application in Point of Care Ultrasound (POCUS)

The ultrasound image is usually obtained and assessed scotopically in the dark. However, the use of sonography at the point of care (POCUS) in emergencies and in the intensive care unit is also important [79]. The need to switch from dark examination rooms to bright diagnostic rooms and lounges is sometimes a challenge due to the limited time available in everyday working life. Here, photopic ultrasound offers qualitative added value compared to the conventional grayscale modality, both in terms of time savings due to the elimination of light-dark adaptation and in terms of improved structure recognition. “Can one claim that there are no stars just because they cannot be seen during the day?”.

## 9. Educational and Interpretive Considerations

Proper interpretation of colorized images requires training. Unlike grayscale imaging, which has universal conventions, photopic imaging depends on device-specific color schemes. Radiologists and sonographers must become familiar with LUT designs and artifact signatures to avoid diagnostic errors.

Educational materials should include

- LUT structure and function
- Examples of typical and atypical color mappings
- Strategies for cross-device interpretation

Case studies and simulation tools can help clinicians become proficient in real-time colorized interpretation.

## 10. Artificial Intelligence Integration and Future Developments

The integration of artificial intelligence (AI) into ultrasound imaging presents a transformative opportunity for photopic (color-tinted) ultrasound, addressing several current limitations while enhancing diagnostic reliability and accessibility. AI-driven approaches can support the real-time optimization, interpretation, and standardization of colorized ultrasound data in ways that go beyond human perception.

### 10.1. AI for Real-Time LUT Optimization

One of the core challenges in daylight sonography is the subjective variability in how Look-Up Tables (LUTs) translate grayscale values into color gradients. AI can help by

- Dynamically adapting LUTs based on anatomical region, tissue type, and ambient light.
- Dynamically adjustment of color schemes to optimize lesion detection or tissue characterization for particular pathologies, thereby providing real-time, disease-tailored visualization
- Learning from expert-reviewed image datasets to optimize contrast and color mapping for specific clinical contexts (e.g., liver lesions, vascular pathology).
- Reducing artifacts such as over-saturation or misleading hue transitions through self-correcting algorithms.
- Future developments may also include disease-specific color adaptation, potentially enabled by AI, to tailor LUTs according to pathology and thereby further enhance diagnostic utility.

### 10.2. Automated Artifact Detection and Suppression

Photopic imaging may introduce subtle visual artifacts unfamiliar to users trained in grayscale interpretation. AI can

- Detect common photopic-specific artifacts (e.g., hole, veiling, or saturation artifacts).
- Provide real-time alerts or automatic correction before image storage or interpretation.
- Improve visual clarity and prevent diagnostic error, especially for novice users.

**Figure 8:**
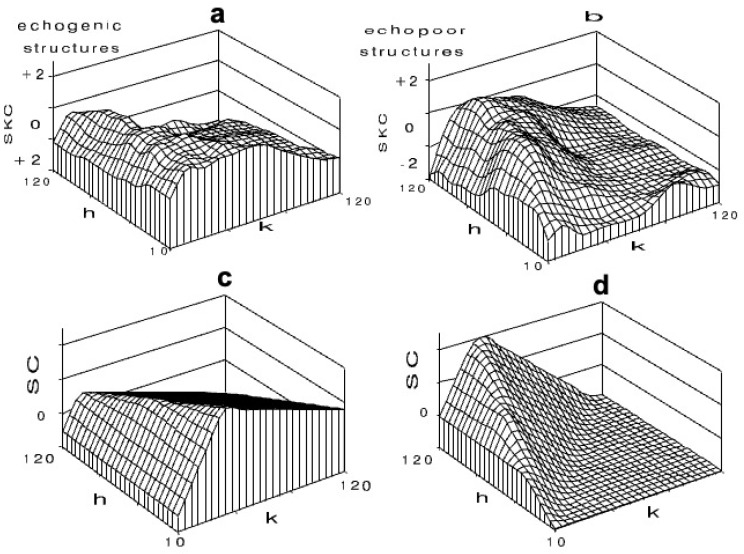
Observations of image changes (SKC) by 7 experienced examiners on an RMI 415 phantom after Photopic transformation with the mean value h and the contrast k, where −2 and −1 were assigned for contrast deterioration, 0 for no change, and +1 to +2 for improvement (**a**,**b**). The computer simulations SC were able to realistically predict these subjective examiner observations (**c**,**d**).

In 1998 Bleck demonstrated in his habilitation thesis a method for automated artifact detection in photopic images using computer-based predicted histogram changes. He was able to show that, in 1008 individual assessments by seven experienced sonographers, the contrast changes resulting from an RMI 415 on a 5-point observation scale (−2 to +2) agreed very well with the computer simulation. This could easily be achieved using an AI algorithm (Figure 8) [2].

### 10.3. Standardization Across Platforms

The diversity of vendor-specific implementations (Photopic Mode, TINT, B-color) creates inconsistency in appearance and interpretation. AI algorithms could

- Translate color mappings across manufacturers for consistent cross-platform imaging.
- Provide vendor-agnostic post-processing that harmonizes image appearance for teaching, research, and telemedicine.

### 10.4. AI-Augmented Education and Simulation

- Color-tinted ultrasound requires tailored training. AI can enhance education through
- Interactive simulators that adapt LUT difficulty based on user proficiency.
- Real-time feedback and scoring during mock exams or image labeling exercises.
- Case-based reasoning systems that help trainees learn from misinterpretations.

### 10.5. Future Perspectives

AI-enhanced daylight sonography could evolve to include [80]:

- Multimodal fusion (e.g., combining photopic B-mode with elastography, CEUS, or Doppler).
- Personalized imaging presets, calibrated to user visual preferences or pathologies of interest.
- Integration into wearable or head-mounted displays for hands-free ultrasound interpretation in emergency or surgical contexts.

## 11. Telemedicine and Remote Consultation

Photopic imaging is especially suited to point-of-care and mobile settings, which benefit from remote expert input. AI can

- Pre-analyze acquired images to flag abnormalities for remote reviewers.
- Compress and transmit optimized, artifact-free colorized images with diagnostic overlays.
- Facilitate global access to expert-level interpretation, especially in low-resource or field environments.

## 12. Teaching

In a practical situation in a bright room, students can better see how the examiner’s right hand handles the probe and what his left hand does with the knobs and buttons of the US machine, and when talking, students and teachers can look each other in the eye. In presentations, colorized US images do not need a dark background, nor does the room need to be as dark as possible, which helps prevent the audience from losing attention or even falling asleep.

## 13. Advantages, Limitations and Future Directions

### 13.1. Advantages of Photopic

Photopic imaging increases both image contrast and brightness while maintaining overall image quality, allowing for better subjective assessment of a very subtle texture difference abnormality. Photopic mode can be used in selected cases as a useful complement to conventional B-mode ultrasound to further enhance image contrast [26,28,63,75,81,82].

By using photopic imaging to electronically colorize B-mode images, we improve the subjective impression of image contrast and increase the adaptability and practicality of B-mode ultrasound in daylight. The improved brightness of the image is particularly advantageous for performing ultrasound under daylight conditions (POCUS, interventional ultrasound, hands-on ultrasound training). The time saved by not having to adapt to the lighting conditions and the improved assessability of the images have clinical (diagnostic, therapeutic and prognostic) implications for POCUS in the emergency or intensive care unit.

Only limited comparative clinical data are currently available [26,28,63,75,81,82]. These results indicate that while photopic imaging has the potential to improve image quality under daylight conditions and clinical outcomes, further large-scale prospective studies are required to establish its statistical superiority in routine practice.

Alternative methods for increasing image brightness, such as higher 2D amplification, are associated with significantly higher background noise and side lobe artifacts. Therefore, photopic imaging leads to improved brightness and higher contrast of an image without being affected by image artifacts.

Improved fatty liver diagnosis can be explained by the enhanced contrast perception and brightness adaptation achieved through photopic imaging. In hepatic steatosis, the echogenicity of liver parenchyma increases relative to renal cortex and diaphragm, often requiring careful adjustment of gain and contrast in conventional grayscale ultrasound. Under daylight conditions, grayscale differences may be subtle and difficult to appreciate. Photopic imaging, by expanding the contrast range and enhancing depth perception, facilitates the visualization of the characteristic brightness changes and improves the discrimination of mild to moderate steatosis. This effect is particularly relevant in routine and point-of-care settings, where examinations are often performed in non-darkened environments.

Another particular advantage of Photopic is the very low transmission power, which is important, for example, in eye ultrasound [78], pregnancy and in obstetrics [83]. Adaptive contrast enhancement is performed in real time, with optimal presentation of the ultrasound data and thus improvements in [1].

- Lower MI and TI with lower transmit power
- Better contrast resolution with lower gain
- Improved visual perception
- Shorter visual reaction times
- Improved depth perception
- Only minor change in gain setting and TGC required
- Better differentiation and identification of small lesions or tissue changes
- No adaptation of the eye to different light conditions required
- Can be used together with THI
- Improved fatty liver diagnostics [84]
- Advantages of CEUS (ECI): Stable vesicles and therefore longer examination time, less contrast medium required, optimally (quantitatively) evaluable CEUS images due to lower saturation during peak amplification.

Different color tones can simulate a spatial depth effect. This phenomenon is already known from Old Master paintings, where “cold colors” such as blue and red are used in the background to make it appear more distant in perspective, while “warm colors” tend to simulate the foreground. It is possible that such a mechanism makes photopic images appear three-dimensional, which makes the images more pleasing and can have a positive effect on the examiner’s ability to concentrate.

### 13.2. Disadvantages of Photopic Imaging

Daylight sonography, despite its advantages, faces several barriers:

- Lack of standardization: Different vendors use non-uniform terms (Photopic Mode, TINT, B-color) and LUT designs.
- Training gaps: Limited formal instruction in color-tinted image interpretation.
- Incomplete validation: Few comparative studies quantitatively assess diagnostic performance, fatigue reduction, or time savings.

Experienced examiners cited the new and unfamiliar nature as a disadvantage, whereas students, i.e., non-influenced examiners, predominantly opted for coloring.

As part of the eye’s dark adaptation, the pupil dilates to allow light to reach the retina. As a result, the ability to focus decreases. However, dark-adapted eyes tend to perceive contrasts better at the edge of the visual field. From this it could be concluded that gray-scale sonography more often detects findings or incidentalomas that are not centrally displayed on the screen. Such an effect would be expected in particular if the examination room is unquestionably completely darkened, and the examiner is therefore dependent on a pronounced dark adaptation of the eyes. This situation should only rarely be the case. However, this hypothesis has not yet been proven.

Current limitations of photopic imaging include the lack of standardized Look-Up Tables across vendors, resulting in variable image appearance and potential interpretation challenges. Training requirements remain underdeveloped, as most sonographers are primarily trained in grayscale imaging. Furthermore, evidence from large multicenter studies is still sparse, so the clinical benefit is not yet fully validated. Finally, photopic imaging may not equally enhance all diagnostic tasks; for example, subtle parenchymal changes may still be better appreciated in grayscale, depending on examiner experience.

## 14. Conclusions

Daylight sonography represents a significant advancement in ultrasound imaging. By aligning visual output with photopic visual physiology, it improves image interpretation, enhances workflow, and expands the use of ultrasound in non-traditional settings. With continued research, standardization, and training, daylight-compatible imaging has the potential to become a new standard in POCUS and beyond.

Important for future development is the reintroduction of Photopic’s original methodology, in which the LUTs were adapted online to the respective image histogram multiple times per second using fast processing, as this represents a significant improvement over static masks. An alternative could be a set of static masks that would be automatically selected based on the ambient brightness in the examination room, determined by a photosensor on the device.

## Figures and Tables

**Table 1:** Some hues optimize visibility under photopic (bright-light) conditions by: Matching the eye’s cone cell sensitivity to color and brightness, enhancing contrast and spatial resolution without introducing veiling or hole artifacts and maintaining diagnostic clarity without requiring a darkened room. Advantages are shown for sepia, blue and green.

Color	Advantages
Sepia (Yellow-Brown)	Mimics familiar tones from black-and-white photography
	Enhances contrast while preserving anatomical detail
	Offers intuitive brightness mapping (lighter tones = more echogenic areas)
	Preferred by students and experts alike
Blue	Provides excellent depth perception
	Reduces visual fatigue by avoiding glare and high saturation
	Often favored by experienced examiners for high-resolution detail
	Facilitates alertness and performance on tasks requiring sustained attention
Green (muted tones)	Offers a balanced hue that avoids excessive saturation
	Enhances intermediate echogenicity levels without causing artifacts
	Especially helpful for liver and parenchymal tissue imaging

## Data Availability

The data presented in this study are available upon request from the corresponding author. Some of the data originates from articles of other authors (see references). The data are not publicly accessible, as the personal rights of the patients involved must be respected.
